# The role of plant labile carbohydrates and nitrogen on wheat-aphid relations 

**DOI:** 10.1038/s41598-021-91424-8

**Published:** 2021-06-15

**Authors:** Victor Sadras, Carolina Vázquez, Elisa Garzo, Aránzazu Moreno, Sonia Medina, Julian Taylor, Alberto Fereres

**Affiliations:** 1 South Australian Research and Development Institute, Adelaide, Australia; 2 School of Agriculture, Food and Wine, The University of Adelaide, Adelaide, Australia; 3 Instituto de Ciencias Agrarias, Consejo Superior de Investigaciones Científicas, ICA‐CSIC, Madrid, Spain; 4 Research Group on Quality, Safety, and Bioactivity of Plant Foods, Department of Food Science and Technology, CEBAS-CSIC, Murcia, Spain

**Keywords:** Entomology, Ecophysiology

## Abstract

Interactions between plants and herbivores are key drivers of evolution and ecosystem complexity. We investigated the role of plant labile carbohydrates and nitrogen on wheat-aphid relations in a 2^2^ factorial combining [CO_2_] and nitrogen supply. We measured life history traits (assay 1) and feeding behaviour (assay 2) of bird-cherry oat aphid (*Rhopalosiphum padi* L.) and English grain aphid (*Sitobion avenae* F.) forced to feed on single leaf laminae, and reproduction of *R. padi* in a setting where insects moved freely along the plant (assay 3). Experimental setting impacted aphid traits. Where aphids were constrained to single leaf, high nitrogen reduced their fitness and discouraged phloem feeding. Where aphids could move throughout the plant, high nitrogen enhanced their reproduction. Aphid responses to the interaction between nitrogen and [CO_2_] varied with experimental setting. The number of *R. padi* adults varied tenfold with plant growing conditions and correlated negatively with molar concentration of sugars in stem (assay 3). This finding has two implications. First, the common interpretation that high nitrogen favours insect fitness because protein-rich animal bodies have to build from nitrogen-poor plant food needs expanding to account for the conspicuous association between low nitrogen and high concentration of labile carbohydrates in plant, which can cause osmotic stress in aphids. Second, the function of labile carbohydrates buffering grain growth needs expanding to account for the osmotic role of carbohydrates in plant resistance to aphids.

## Introduction

Interactions between organisms are major drivers of evolution and ecosystem complexity^1–5^. The decupling of trophic webs is a conspicuous, ecologically and agronomically significant effect of global change^6–9^. Air CO_2_ concentration [CO_2_], ambient temperature, availability of water and nitrogen influence the plant phenotype with bottom-up consequences for the behaviour and fitness of herbivorous arthropods^9–13^.

Multiple stresses are often non-additive and hence largely unpredictable^1–4^. Waring and Cobb^10^ highlight the difficulty in predicting sign and magnitude of interactions, with significant interactions—whether positive, negative, or non-linear—in 338 out of 450 cases for herbivores feeding on water- or nutrient-stressed plants. Elevated [CO_2_], high temperature and nitrogen deficit alter plant nutritional quality and secondary metabolites^9,11–13^. However, the impact of elevated [CO_2_] interacting with temperature or nitrogen on insects, mediated by changes in plant phenotype, cannot be deduced from the effects of the individual factors^13,14^. Furthermore, insect responses to host plants under elevated [CO_2_] are guild-dependent^12^.

Wheat, which contributes about 20% of the total dietary calories and proteins worldwide, is critical to food security^15^ and aphids (Hemiptera, Aphidoidea) together with the viruses they transmit are its most important pests^16–18^. *Rhopalosiphum padi* and *Sitobion avenae,* the focus of this study, are common pests of wheat worldwide with distinct niches whereby *R. padi* prefers the stem and basal leaves whilst *S. avenae* prefers the upper leaves and ears^19^.

Lagging theory is a bottleneck to understand the phenotype of plants and animals^20–22^. We have recently advanced a conceptual model that emphasises a dual role of labile carbohydrates in cereals: in the carbon economy, buffering grain growth, and in plant defence, disrupting osmoregulation in aphids^23^. The concentration of nitrogen in plants correlates negatively with the concentration of labile carbohydrates^23^. Typically, plants grown under elevated [CO_2_] feature lower nitrogen content and higher carbohydrate content than their counterparts under low [CO_2_]^12,14^.

Here we report the shifts in life-history and feeding behaviour of *Rhopalosiphum padi* and *Sitobion avenae* in response to changes in the profiles of labile carbohydrates and amino acids of wheat plants grown under the factorial combination of two [CO_2_] and two rates of nitrogen supply. Furthermore, we studied how aphid reproduction varies with the part of the plant where they are allowed to feed.

## Results

### Wheat growth in response to nitrogen and [CO_2_]

**Figure 1:**
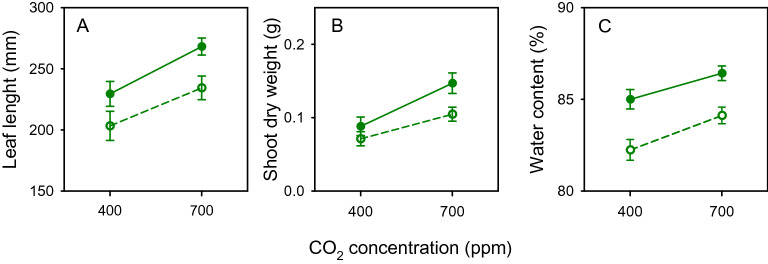
Effects of nitrogen (high: closed symbol, low: open symbol) and [CO_2_] on the (**A**) length of the third leaf of wheat plants at 21 DAS, and (**B**) shoot dry weight and (**C**) shoot water content at 28 DAS. Error bars are two standard errors of the mean.

Nominal nitrogen treatments can fail to cause variation in plant growth^24^. Thus, we measured leaf length twice a week to detect the onset of nitrogen and [CO_2_] effects on the host plant before establishing aphid assays; leaf expansion is particularly sensitive to nitrogen deficit^25^. At 21 days after sowing (DAS), we recorded treatment effects for the first time: leaves were longer under high nitrogen (*p* = 0.0032, *s* = 8.3) and high [CO_2_] (*p* = 0.0007, *s* = 10.5), with no interaction between nitrogen and [CO_2_] (*p* = 0.70, *s* = 0.5) (Fig. 1A). Dry matter also increased additively (interaction *p* = 0.31, *s* = 1.7) with high nitrogen (*p* = 0.022, *s* = 5.5) and high [CO_2_] (*p* = 0.0009, *s* = 10.1) (Fig. 1B). Percentage of water in shoot increased additively (interaction *p* = 0.64, *s* = 0.6) with high nitrogen (*p* < 0.0001, *s* > 13.3) and high [CO_2_] (*p* = 0.027, *s* = 8.5) (Fig. 1C).

### Sugars and amino acids in plant

Plant age spanned from 28 days at the beginning of aphid assays 1 and 2 to 49 days at the end of assay 3. Hence, we sampled plants at both ages to account for ontogenetic effects on composition.

**Figure 2:**
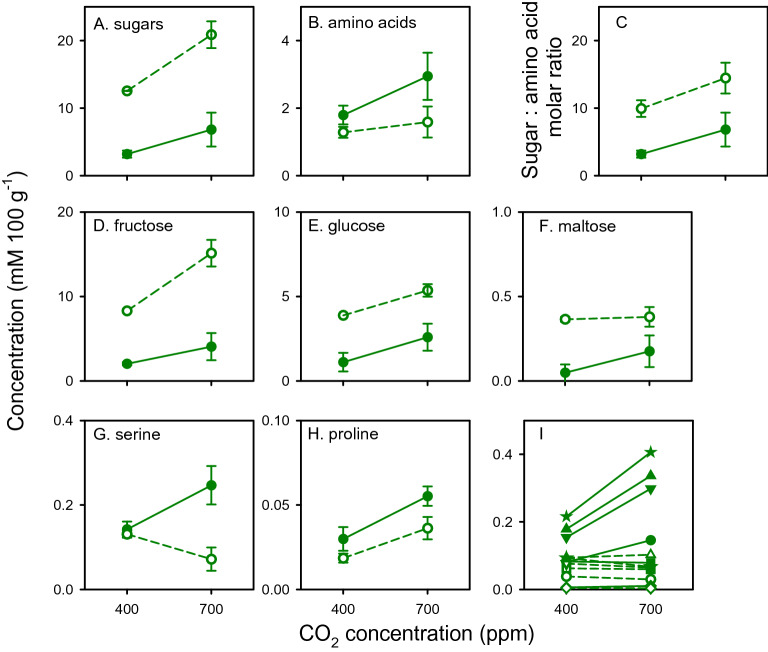
Effects of nitrogen (high: closed symbol, low: open symbol), [CO_2_] and their interaction on the concentration of sugars and amino acids in wheat plants at 28 days after sowing, immediately before the establishment of aphid assays 1 and 2. (**A**) Total sugars, (**B**) total amino acids, (**C**) sugar: amino acid molar ratio, (**D**) fructose, (**E**) glucose, (**F**) maltose. (**G**) Serine, which varied with nitrogen and interaction. (**H**) Proline, which varied with nitrogen and [CO_2_], with no interaction. (**I**) Examples of amino acids that increased concentration with high nitrogen and did not vary with [CO_2_] and interaction: circles, valine; squares, arginine; triangles, amino butyric acid; triangles down, phenylalanine; lozenges, threonine; stars, leucine. Error bars are two standard errors of the mean and have been omitted for clarity in I.

Figure 2 shows the effects of nitrogen, [CO_2_] and their interaction on the concentration of sugars and amino acids in plants at 28 DAS, immediately before the establishment of aphid assays 1 and 2; Supplementary Table 1 presents *p* and *s* from linear mixed model analysis. Low nitrogen (*p* < 0.0001, *s* = 50.2) and high [CO_2_] (*p* < 0.0001 s = 21.2) increased the total concentration of sugars additively (interaction: *p* = 0.07, *s* = 3.9) (Fig. 2A). Total concentration of amino acids increased with high nitrogen (*p* = 0.039, *s* = 4.7) and did not vary with [CO_2_] (*p* = 0.25, *s* = 2.0) or interaction (*p* = 0.52, *s* = 0.9) (Fig. 2B). The molar ratio of sugars to amino acids increased additively (interaction *p* = 0.36, *s* = 1.5) with low nitrogen (*p* < 0.0001, *s* = 27.2) and high [CO_2_] (*p* = 0.028, *s* = 5.2) (Fig. 2C).

The concentration of sugars ranked fructose > glucose > maltose, with sucrose below detection level (Fig. 2D–F). Plants grown with high nitrogen had lower concentration of fructose, glucose and maltose than their counterparts with low nitrogen (Fig. 2D–F, Supplementary Table 1). Fructose and glucose, but not maltose, increased with high [CO_2_] (Fig. 2D–F). The increase in fructose concentration with high [CO_2_] was larger under low nitrogen (interaction *p* = 0.01, *s* = 6.7) (Fig. 2D); glucose and maltose did not vary with the interaction between [CO_2_] and nitrogen (Supplementary Table 1).

Three amino acids were unresponsive to all three sources of variation: glycine, glutamic acid, and tyrosine (Supplementary Table 1). Serine varied with both nitrogen (*p* = 0.001, *s* = 9.5) and the interaction between nitrogen and [CO_2_] (*p* = 0.004, *s* = 8.1), increasing concentration in response to high nitrogen at high [CO_2_] and decreasing concentration in response to low nitrogen at high [CO_2_] (Fig. 2G, Supplementary Table 1). Proline increased with both high nitrogen (*p* = 0.012, *s* = 6.4) and high [CO_2_] (*p* = 0.001, *s* = 11.1), with no response to interaction (*p* = 0.99, *s* = 0.0) (Fig. 2H). Amino butyric acid, leucine, phenylalanine, valine, arginine, threonine, alanine and hydroxyproline all increased with high nitrogen, and were unresponsive to both [CO_2_] and interaction (Fig. 2I, Supplementary Table 1).

**Figure 3:**
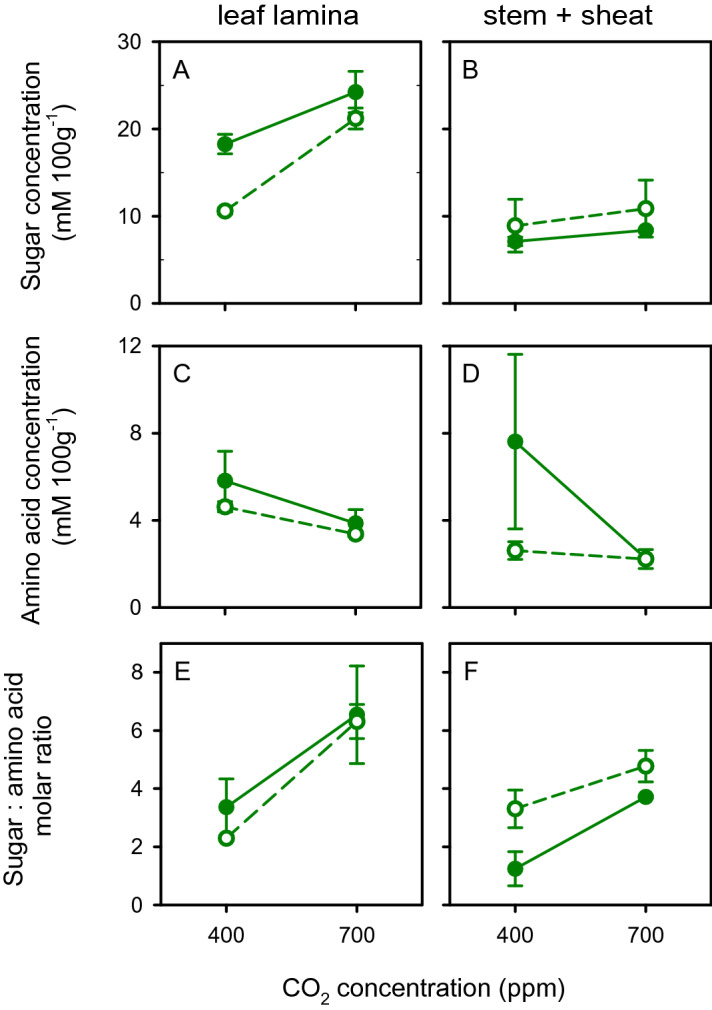
Effects of nitrogen (high: closed symbol, low: open symbol), [CO_2_] and their interaction on the concentration of sugars and amino acids on wheat plants at 49 days after sowing, immediately after the completion of aphid assay 3. (**A**, **B**) Total sugars, (**C**, **D**) total amino acids, (**E**, **F**) sugar: amino acid molar ratio in (**A**, **C**, **E**) leaf lamina and (**B**, **D**, **F**) stem + sheath. Error bars are two standard errors of the mean.

Figure 3 shows the effects of nitrogen, [CO_2_] and their interaction on the concentration of sugars and amino acids in plants at 49 DAS, immediately after the completion of aphid assay 3. The total concentration of sugars increased with high [CO_2_] (*p* = 0.0059, *s* = 7.4) and was larger in leaf than in stem (*p* < 0.0001, *s* > 13.3). The increase in sugar concentration with high [CO_2_] was larger in leaf than in stem (interaction plant part by [CO_2_]: *p* = 0.037, *s* = 4.8). The concentration of sugars was reduced in leaf and increased in stem in high nitrogen plants compared with their low nitrogen counterparts (interaction plant part by nitrogen: *p* = 0.023, *s* = 5.4). Treatments did not affect the total concentration of amino acids; hence, the molar ratio of sugars to amino acids was higher in leaf than in stem (*p* = 0.042, *s* = 4.6), doubled with high [CO_2_] (*p* = 0.0012, *s* = 9.7) and did not vary with nitrogen (*p* = 0.44, *s* = 1.2) or interaction.

### Assay 1: Aphid life-history traits on single leaf

**Figure 4:**
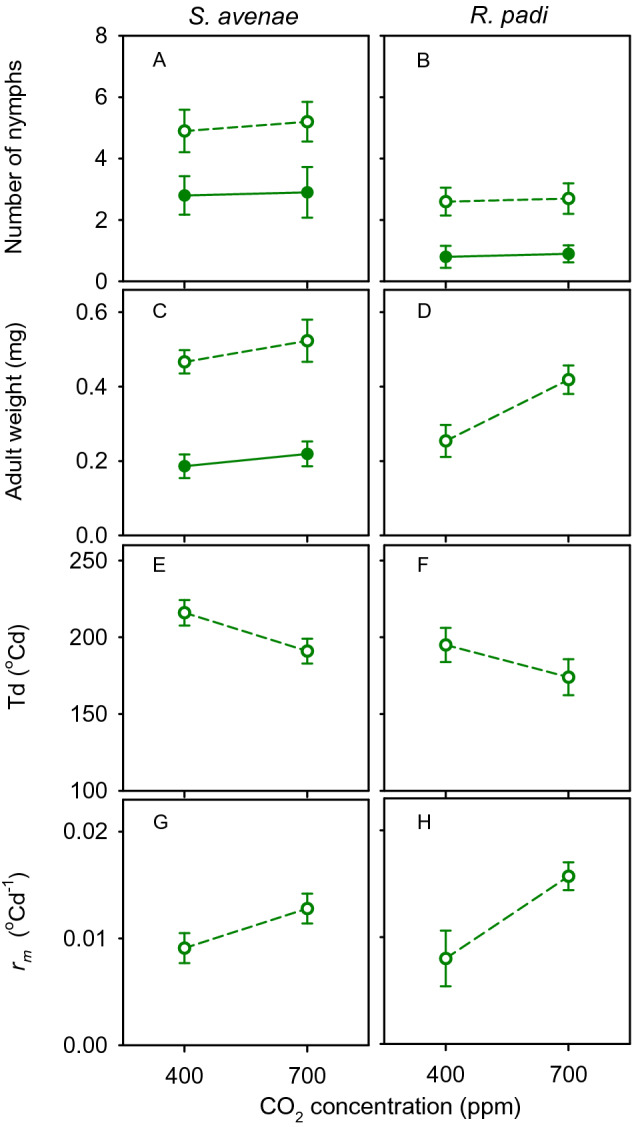
(**A**, **B**) Number of *S. avena*e and* R. padi *nymphs laid by a 2*–*3 d old adult over 48 h on wheat leaves in response to nitrogen supply (high: closed symbol, low: open symbol), [CO_2_] and their interaction. (**C**, **D**) Weight of adults at the onset of reproduction; no* R. padi* survived to adulthood under high nitrogen. (**E**, **F**) generational time *Td* and (G, H) intrinsic rate of natural increase* ﻿r*_m_ for* R. padi* and *S. avenae* on wheat leaves under low nitrogen and two [CO_2_]. Error bars are two standard errors of the mean.

In a clip-cage assay^26^, a 2–3-day old adult was placed on the third leaf of each test plant at 28 DAS. After 48 h, *S. avenae* had laid more nymphs than *R. padi* (*p* < 0.0001, *s* > 13.3), and high nitrogen reduced the number of nymphs in relation to their counterparts under low nitrogen in both *S. avenae* (*p* = 0.006, *s* = 7.3) and *R. padi* (*p* < 0.0001, *s* > 13.3), with no effect of [CO_2_] (*p* = 0.57, *s* = 0.8) or interaction between nitrogen and [CO_2_] (*p* = 0.57, *s* = 0.8) (Fig. 4A, B).

Of these newly born *R. padi* nymphs, none survived on leaves with high nitrogen. *Sitobion avenae* nymphs survived to reproductive stage irrespective of treatment, but high nitrogen halved their weight in relation to those raised in low nitrogen leaves (*p* < 0.0001, *s* > 13.3) (Fig. 4C). Adult weight at the onset of reproduction did not change with [CO_2_] (*p* = 0.26, *s* = 1.9) or interaction between nitrogen and [CO_2_] (*p* = 0.76, *s* = 0.4) in *S. avenae* (Fig. 4C), whereas *R. padi* adults raised in plants under high [CO_2_] were 40% heavier than their counterparts under low [CO_2_] (Fig. 4D; *p* = 0.013, *s* = 6.3). Owing to the extreme effect of elevated nitrogen, the analysis of life-history traits in assay 1 was restricted to the low nitrogen treatment (Fig. 4E–H). In relation to ambient [CO_2_], elevated [CO_2_] shortened generational time (Eq. 1) by 11% in both species (Fig. 4E, F; *p* = 0.032, *s* = 5.0) and enhanced the intrinsic rate of natural increase (Eq. 2) twofold in *R. padi* and 30% in *S. avenae* (Fig. 4G, H; *p* = 0.0002, *s* = 9.0).

### Assay 2: Aphid feeding behaviour on single leaf

#### Overview

**Figure 5:**
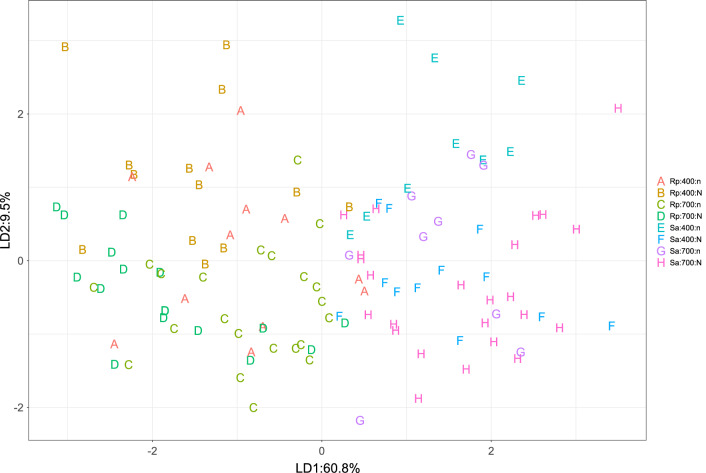
First two linear discriminants of the multivariate EPG data for the complete set of samples (16 ≤ n ≤ 19) across the combination of nitrogen (n, low; N, high), [CO_2_] (400 and 700 ppm) and aphid species (Rp, *Rhopalosiphum padi;* Sa, *Sitobion avenae*). The first linear discriminant explained 60.8% of the variation and the second discriminant explained 9.5%. The multivariate EPG data comprises 19 variables, after accounting for both non-zero values and redundancy in the complete set of variables.

The feeding behaviour of *S. avenae* and *R. padi* in plants grown under the 2^2^ factorial combining nitrogen and [CO_2_] was evaluated in 8-h EPG tests (Supplementary Tables 2 and 3). We performed a discriminant analysis to capture the effects of the full combination of experimental sources of variation—nitrogen, [CO_2_], aphid species—on the multivariate EPG data. Aphid species clustered along the first linear discriminant (Fig. 5). The first linear discriminant also separated nitrogen effects for *R. padi*. The second linear discriminant separated [CO_2_] effects (Fig. 5).

#### Dynamics of aphid passive feeding on phloem sap (waveform E2)

**Figure 6:**
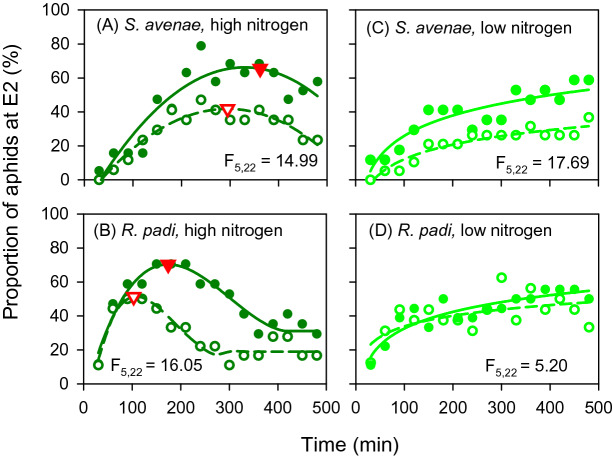
Proportion of aphids passively feeding on phloem sap (waveform E2) as affected by growing conditions of wheat plants in 8-h EPG assays. Open symbols are low [CO_2_] and closed symbols are high [CO_2_]. Red arrowheads in (**A**, **B**) show maxima. Polynomials were fitted to high nitrogen (0.55 < R^2^ < 0.90) and logarithmic functions to low nitrogen (0.39 < R^2^ < 0.90). An F-test for pair-wise comparison of fitted curves returned *p* < 0.00001, s > 16.6 for (**A**, **B**, **C**) and *p* = 0.003, s = 8.4 for (**D**).

Averages (Supplementary Tables 2 and 3) and discriminant analysis (Fig. 5) are static. Figure 6 adds a time dimension to the biologically relevant waveform *E2* representing passive feeding on phloem sap^27,28^. Irrespective of aphid species and [CO_2_], high nitrogen discouraged phloem feeding: the proportion of aphids at *E2* in the high nitrogen treatment increased initially, reached a peak, and declined at later stages of the assay (Fig. 6A, B). The rejection of high nitrogen plants was sharper in *R. padi,* which peaked at 103–174 min, compared to *S. avenae,* which peaked at 295–362 min (red arrow heads in Fig. 4A, B). The different reaction between aphid species was also apparent in the lower proportion of *R. padi* feeding on high nitrogen plants at the end of the assay*.* In contrast, the proportion of aphids at *E2* increased at diminishing rates but showed no peak or decline under low nitrogen (Fig. 6C, D). Comparisons of curves showed variation with [CO_2_] treatment for both *S. avenae* (F_5,22_ = 14.99, *p* < 0.00001, *s* > 16.6) and *R. padi* (F_5,22_ = 16.05, *p* < 0.00001, *s* > 16.6) under high nitrogen (Fig. 6A, B). This was reflected in delayed and higher peak for the proportion of aphids at *E2* (red arrowheads in Fig. 6A, B) at elevated [CO_2_] compared with low [CO_2_]. [CO_2_] under low nitrogen affected both aphid species, but the effect was stronger for *S. avenae* (F_5,22_ = 21.80, *p* < 0.0001, *s* > 16.6) than for *R. padi* (F_5,22_ = 5.20, *p* = 0.003, *s* = 8.4).

### Assay 3: aphid reproduction on whole plant

**Figure 7:**
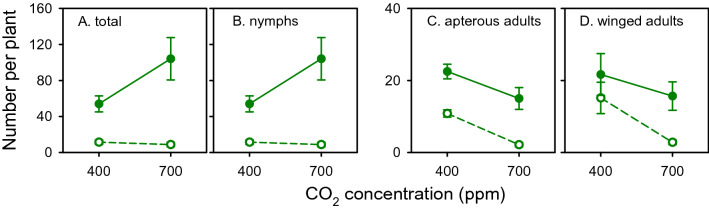
Effect of nitrogen (high: closed symbol, low: open symbol), [CO_2_] and their interaction on the number *Rophalosiphon padi* in whole-plant assay 3. (**A**) Total number of aphids, (**B**) nymphs, (**C**) apterous adults, and (**D**) winged adults. Error bars are two standard errors of the mean.

**Figure 8:**
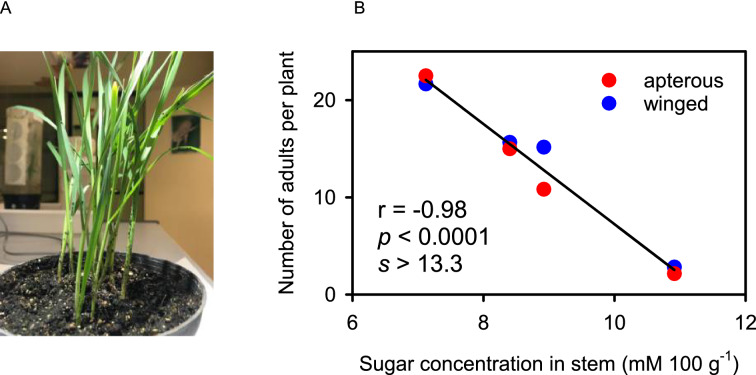
(**A**) *Rophalosiphon padi* established and fed preferentially in the basal part of the plant. (**B**) Number of *R. padi* adults (winged and apterous) declined with increasing molar concentration of sugars in wheat stem, assay 3. The line is the least-square regression.

*Rhopalosiphum padi* was particularly sensitive to high nitrogen in assays 1 and 2, where insects were forced to single leaves. We thus tested *R. padi* reproduction in response to nitrogen and [CO_2_] in assay 3, where insects were allowed to move freely along the plant. High nitrogen enhanced reproduction of *R. padi* (*p* < 0.0001, *s* > 13.3)*,* with a stronger effect on total number of insects at high [CO_2_] (interaction *p* = 0.005, *s* = 7.6) (Fig. 7A). We did not measure, but observed, a larger proportion of aphids on the basal part of plants (Fig. 8A), consistent with independent studies showing the proportion of *R. padi* increased from 13% at the top of the plant, to 20% in the middle, to 67% at the bottom^19^. Total number of adults correlated negatively with the molar concentration of sugars in stem (Fig. 8B).

## Discussion

### Effects of nitrogen and [CO_2_] on aphid fitness and behaviour

Insect fitness is often diminished in nitrogen-deficient host plants^29–31^, but there are exceptions. In a sample of 85 studies, 58% showed nitrogen fertilisation was beneficial for sucking insects, 13% returned negative or non-linear effects with no nitrogen effect for the remaining cases^10^. In an experiment with three related plant species, only *Miscanthus sinensis* biomass responded to nitrogen fertiliser, and high nitrogen supply reduced the intrinsic rate of population increase of *Rhopalosiphum maidis*^24^. Similarly, high nitrogen supply was detrimental for both aphid species in assays 1 and 2, where insects were forced to feed on single leaf laminae (Figs. 4 and 6). The time-trajectory of the waveform *E2* (phloem sap ingestion) in assay 2 indicated aphids rejected high-nitrogen plants after a transient increase in the proportion of aphids passively feeding on phloem sap (Fig. 6). The rejection of high nitrogen plants was particularly sharp in *R. padi*. In contrast, where aphids were able to move along the plant, high nitrogen favoured reproduction (Fig. 7).

Many experiments, including ours, are based on individual plants where “high” or “low” nitrogen are nominal treatments, with no objective measure of plant nitrogen status necessary for meaningful comparisons. Owing to the allometric dilution of nitrogen with plant biomass, nitrogen concentration is a misleading indicator of plant nitrogen status^32,33^. In crop stands, nitrogen status is quantified unequivocally with the nitrogen nutrition index, calculated as the ratio of actual nitrogen concentration and a critical nitrogen concentration from robust dilution curves^32,33^. However, nitrogen dilution curves differ between isolated individual plants and plants in stands^34^. Methods to rigorously quantify nitrogen status of individual plants are required.

The effect of elevated [CO_2_] on aphid fitness and behaviour is idiosyncratic—it depends on the specific aphid-plant system and background experimental conditions including the actual concentrations of CO_2_ in the elevated and control treatments, and other factors such as temperature^35^. Our comparison of assays also shows the influence of experimental method to measure insect traits. Elevated [CO_2_] improved fitness of *Aphis gossypii*, *S. avenae* and *Acyrthosiphon pisum* on barrel medic and of *R. padi* on wheat^36–38^. Conversely, host pepper plants grown at [CO_2_] = 650 ppm extended *Myzus persicae* pre-reproductive period and reduced its fecundity in comparison to host plants grown at [CO_2_] = 400 ppm^39^. In plants grown at elevated [CO_2_], *M. persicae* decreased salivation into sieve elements (*E1*) with no differences in phloem ingestion (*E2*). The body mass of *A gossypii* decreased when feeding on melon plants acclimated for 6 weeks under [CO_2_] = 800 ppm and 22 °C compared to [CO_2_] = 400 ppm and 20 °C^14^. We found elevated [CO_2_] enhanced fitness of both aphid species under low nitrogen in single leaf assay 1 (Fig. 4). Further, elevated [CO_2_] enhanced passive feeding on phloem sap irrespective of nitrogen treatment (Fig. 6). In assay 3, where aphids were free to move, high [CO_2_] enhanced reproduction of *R. padi* under high but not under low nitrogen (Fig. 7). Under the experimental conditions of Navarro et al.^13^, *R. padi* fecundity measured in a setting similar to our assay 1 declined in wheat grown under [CO_2_] = 800 ppm compared to controls at 400 ppm and high nitrogen supply partially offset the effect of elevated [CO_2_].

### Effects of nitrogen and [CO_2_] on plant composition and implications for aphid fitness and behaviour

C:N is a stoichiometric ratio commonly used to assess plant suitability to herbivores. High C:N in plant tissue is a conspicuous effect of elevated [CO_2_]^40^ and has been invoked to explain the reduction in fitness of insects on host plants grown under elevated [CO_2_] as well as for the reduction of insect fitness in nitrogen-deficient plants^14,39^. However, high C:N cannot account for the many cases where elevated [CO_2_] favours insects nor the exceptions where high nitrogen supply is detrimental to insects, as discussed in the previous section. Moreover, C:N measured on whole leaves or plants is not necessarily relevant to phloem-feeding insects. Nitrogen concentration in wheat shoot ranges from 1 to 5%^41^ and is mostly associated with photosynthetic proteins^42^ whereas the concentration of nitrogen in phloem sap is 0.004 to 0.6%^29^ and is primarily associated with amino acids^37^. Hence, we focused on sugars and amino acids to probe for the effects of plant growing conditions on aphid fitness and behaviour.

Elevated [CO_2_] increased leaf concentration of fructose and glucose in our study (Fig. 2). Similarly, increasing [CO_2_] from 400 to 600 ppm increased content of fructose and glucose on wheat phloem sap^37^. The increase in sugar concentration in our study was 5-times larger than that reported by Oehme et al.^37^. This partially reflects the difference in elevated [CO_2_], i.e., 700 ppm in our study versus 600 ppm in theirs, but more likely differences arising from sampling whole tissue versus phloem.

The response of aphids to sugars in plant is likely non-linear, with aphid fitness impaired by reduced feeding reflecting the importance of sugars as phagostimulants at low dietary sugar concentrations, and by osmoregulation failure at high concentrations^23,27,43,44^. Consistently with the widely documented negative correlation between nitrogen and labile carbohydrates in plants^23^, we found concentration of sugars declined with increasing nitrogen (Figs. 3 and 4). High nitrogen reduced the concentration of sugars proportionally more than it increased concentration of amino acids, hence the fivefold increase in the molar ratio of sugars to amino acids in plants with low nitrogen supply compared to their counterparts with high nitrogen. Concentration of sugars in stem accounted for 98% of the variation in adult number in assay 3 (Fig. 8), consistent with the deleterious osmotic effect of sugars on aphid fitness^23,27,44^. A comparative study showed *R. padi* prefers stems and basal leaves of wheat plants whereas *S. avenae* favours the upper leaves and ears, and these preferences were partially attributed to their differential adaptations to microclimate and plant composition^19^. As the concentration of osmotically active sugars was lower in stem than in leaf lamina (Fig. 3), we speculate that *R. padi* preference for stems might be a behavioural adaptation to osmotic stress whereas *S. avenae* might be better able to cope with higher concentration of sugars in leaves, for example by diluting phloem sap with increased uptake of xylem sap^27^. Experiments are needed to directly compare osmoregulation in both aphid species.

## Conclusion

Experimental setting had a large impact on aphids feeding on wheat plants grown under contrasting nitrogen supply and [CO_2_]. Where insects were constrained to single leaf laminae in fitness and feeding assays, high nitrogen was deleterious. Where insects where allowed to move, high nitrogen favoured aphid reproduction as they were able to select the more suitable feeding site within the plant. Clip-cage fitness assays and EPG feeding studies could be adapted to stems to accommodate insect preferences as needed. The C:N ratio is commonly used to evaluate response of herbivores to plants, but labile forms, i.e. sugars and amino acids, are more relevant for sucking insects.

A lagging theory of the phenotype^20–22^ limits our understanding of plants, insects and their interactions. Our study has theoretical implications for wheat-aphid relations, and for the dual role of labile carbohydrates (i.e., reserve and osmotic) in the phenotype of wheat. The common interpretation that high nitrogen favours insect fitness because protein-rich animal bodies have to build from nitrogen-poor plant food needs to be expanded to account for the effect of nitrogen mediated by labile carbohydrates: lower plant nitrogen associates with higher concentration of labile carbohydrates, which can cause osmotic stress in aphids. Thus, plants grown under low nitrogen supply could be detrimental to aphids not only because of the reduction of certain amino acids in the phloem sap but also because of the high concentration of osmotically active sugars. Physiological, ecological and agronomic studies mostly focus on the role of labile carbohydrates in the carbon economy of the plant, for example supporting growth after herbivory and grain fill under stress^45–48^. The common interpretation of water-soluble carbohydrates playing a buffering role for grain growth in wheat thus needs expanding to account for their osmotic role in plant defence against aphids.

## Methods

All methods were performed following the relevant guidelines and regulations.

### Plant growth in a nitrogen × [CO_2_] factorial

Wheat (cv Pedrosa) plants were grown in 9 × 9 × 10 cm pots with vermiculite (Asfaltex S.A., Barcelona, Spain) in growth chambers. A factorial combining two [CO_2_] (ambient: 400 ppm, elevated: 700 ppm) and two nitrogen rates returned four treatments. Eight days after sowing (DAS), two nitrogen treatments were established whereby plants were watered with either full Hoagland solution (high nitrogen) or with Hoagland solution were nitrogen was reduced to 20% of the full solution (low nitrogen); nutrient solution was applied three times a week in both treatments.

Day:night cycles were 14: 10 h, with three Philips Green Power LED Production Modules Deep Red/Blue 150 providing 200 µmol m^−2^ s^−1^ at canopy level. Day temperature was 20.7 ± 0.01 °C and night temperature was 6.2 ± 0.02 °C and vapour pressure deficit was 0.53 ± 0.005 kPa and 0.27 ± 0.003 kPa. There was a slight difference in temperature between chambers, with daily mean temperature 19.1 ± 0.10 °C in the high [CO_2_] chamber and 18.6 ± 0.09 °C in the ambient [CO_2_] chamber. Thermal time was used to account for this temperature difference in the calculation of aphid life-history traits (see below). Twice-weekly measurements of leaf length were used to detect the onset of treatment effects to inform the decision on the start of assays 1 and 2 (see below).

### Aphid assays

We performed two independent assays with both *Rhophalosiphum padi* and *Sitobion avenae* (assays 1 and 2) and a third assay with *R. padi* (assay 3). *Rhophalosiphum padi* colony was generated from a single virginoparous female collected in 2013 from a barley plant (*Hordeum vulgare*) in La Poveda Experimental Farm (Madrid, Spain). *Sitobion avenae* population was supplied by Koppert Biological Systems in 2013. Both populations were maintained on barley in a growth chamber with 14 h photoperiod and day:night temperature of 22 °C:18 °C.

### Assay 1: Fitness of *R. padi* and *S. avenae* confined on single leaves

We evaluated life-history traits of both aphid species on plants grown in the factorial combination of [CO_2_] and nitrogen supply (n = 10). Apterous adults from aphid colonies of approximately equal age and weight were used. For synchronisation, and to obtain adults of the same age, three apterous adult females were placed inside a plexiglass cage (9 × 6 × 1.5 cm) with barley leaves that were replaced every 2 days. The following day, adults were removed and newly born nymphs were kept in plexiglass cages (restricted movement) on the 3^rd^ leaf. Nymphs reached adulthood 7–8 days later. A 2–3-day old adult was placed on each test plant using a clip-cage designed to contain cereal leaves as described in Fereres et al.^26^. After 24 h each adult was removed from the test plant, and 2 newly born nymphs were left inside each clip-cage. Nymphs were allowed to develop inside each clip cage until they reached the reproductive stage. At that point, a single adult was left inside each clip cage and nymph survivorship and developmental time was recorded. The newly born nymphs per adult were counted every 24 h and removed to avoid crowding effects that could influence the reproductive potential. The assay continued until adults reached the time equivalent to the pre-reproductive period (between 8 and 14 d depending on treatment). According to the assumptions and models summarised by Dixon^49^, we calculated the generational time *Td* and the intrinsic rate of natural increase of the aphid population *r*_*m*_:

1 $$Td = d/0.{738}$$

2 $$r_{m} = 0.{738 }\left( {{\text{ln}}\;Nd} \right)/d$$

where *d* is the pre-reproductive period, from birth to onset of reproduction, and *Nd* is the number of offspring produced by an adult during *d* days. To account for the slight difference in daily mean temperature between the chambers (see above), *d* was calculated as thermal time above a base temperature of 5 °C^50^, hence *Td* is reported in °Cd, and *r*_*m*_ in °Cd^−1^.

### Assay 2: Feeding behaviour of *R. padi* and *S. avenae*

The feeding behaviour assay was initiated 28 DAS, at the onset of detectable effects of [CO_2_] and nitrogen on wheat plants. *R. padi* and *S. avenae* were allowed to feed on the youngest fully expanded leaf of test plants for 8 h once they were connected to the EPG device (EPG Systems, Wageningen, The Netherlands). A minimum of 16 replicates were made for each combination of [CO_2_], nitrogen and aphid species. A different single aphid and single plant were used for each replicate. Apterous adult aphids were immobilised with a vacuum device, and a gold wire (2 cm length, 18.5 μm diameter (EPG Systems)) was attached to the aphid dorsum using hand-mixed, water-based silver conductive paint glue (EPG Systems). Then, the opposite end of the gold wire was soldered to the brass insect pin that was inserted into the input connector of the EPG probe. Another copper electrode (10 cm length, 2 mm diameter) was inserted into the soil of the plant container. One Giga-4 and one Giga-8 DC-EPG device with 1 GΩ input resistance (EPG Systems) were used to monitor the probing and feeding behaviour of aphids on plants. The EPG output was set to 50 × gain and data were acquired at 100 Hz. The EPG data were acquired through a USB analog/digital converter card (DI-155 and DI-710; DATAQ Instruments, Akron, OH) using Stylet + software (EPG Systems) and stored in a PC. All EPG recordings were conducted under laboratory conditions at 25 ± 2 °C inside a Faraday cage to avoid electrical noise and interferences.

EPG data were analysed using Stylet + software for Windows (EPG Systems). EPG waveforms associated with specific stylet tip positions and activities when aphids probed and fed on plants were: *np*, non-probing behaviour, and *C*, which represents the intercellular apoplastic stylet pathway where the insects show a cyclic activity of mechanical stylet penetration and secretion of saliva; *E1*, which represents salivation into phloem sieve elements at the beginning of the phloem phase; *E2*, passive phloem sap uptake; *G*, which represents active xylem sap intake, and waveform *F*, related to derailed stylet mechanics.

### Assay 3: Reproduction of *R. padi* on whole plants

Assays 1 and 2, where insects were forced to feed on a single leaf, showed *R. padi* was particularly sensitive to nitrogen treatment. Further, *R. padi* prefers to settle in the lower part of wheat plants^19^. Thus, we conducted assay 3 to quantify the reproduction of *R. padi* in a setting where insects were free to move along the plant. The experiment was the full factorial of two nitrogen and two [CO_2_] regimes described above, with six replicates per treatment. At 35 DAS, four adults of the same age were placed on the third leaf of each plant. Polyethylene non-woven textile (Reicrop 17™, Texnovo, Barcelona, Spain) supported with a purpose-built frame was used to cover each individual plant, allowing aphids to move freely along the plant. This material transmits 80% of light, allows for air flow and is used to protect crops from insect pests; further details of this method are in Cid et al.^51^. Growing conditions described above were maintained during the 2-week duration of assay 3. After 2 weeks, we counted the adults (apterae and alate) and nymphs on each plant.

### Carbohydrates and amino acids in plant

#### Sampling

To explore the relations between aphid traits and plant composition, plants were sampled before assays 1 and 2 and immediately after assay 3. Samples included: (1) three composite samples of 2–3 shoots per treatment at 28 DAS, immediately before aphid assays 1 and 2; and (2) three composite samples, each comprising 3 shoots separated in stem + sheath and lamina after assay 3. Plant tissues were stored at − 80 °C immediately after sampling.

#### Amino acids

We homogenised 2-g samples in freeze MeOH 80% (1:2 w/v) with a blender operated at 1200 W for 1 min, and analysed free amino acid content with the method by Collado-Gonzalez et al.^52^ and Riga et al.^53^. The extraction was carried out with 12.5 µL of MeOH/water (50%, v/v) for an aliquot of 500 µL of sample. Then, these solutions were vortexed for 30 s on ice followed by incubation on ice for 5 min and a sonication in an ultrasound bath for 1 min. The homogenates were centrifuged (centrifuge 5804 R, Hamburg, Germany) for 10 min at 17,900 g and 4 °C. The supernatant was transferred to a limited volume vial and extracts were derivatised immediately with the 6-aminoquinolyl-N-hydroxysuccinimidyl-carbamate (AQC) as derivatising reagent, which can react with primary or secondary amine producing the 6-aminoquinoline (AMQ) derivatives and N-hydroxysuccinimide (NHS)^52^. The derivatisation was carried out with the method by Nagumo et al.^54^.

Chromatographic analyses for the identification and quantification were carried out on an AccQ Tag Ultra BEH column (2.1 × 100 mm, 1.7 µm) (Waters corp., Ireland); 50 mL of an aqueous solution (acetonitrile, formic acid, and acetate ammonium in water [5 mM] 10:6:84, v/v/v) diluted with 950 mL ultrapure water and acetonitrile/formic acid (99.9:0.1, v/v) were used for chromatographic separation in an UHPLC system coupled with a 6460 tandem mass spectrometer (Agilent Technologies, Waldbronn, Germany) with same working conditions for the MS parameters, MS parameters fragmentor and collision energy described by Collado-Gonzalez et al.^52^ and Cerrillo et al.^55^. We used MassHunter software v. B.04.00 (Agilent Technologies) for data acquisition and processing. 20-μL of derivatised amino acid standards or samples were injected onto the column and eluted at flow rate of 0.5 mL min^−1^. The acquisition time was 12 min for each standard or sample^53^. The MS analysis was applied in the multiple reaction monitoring (MRM) mode, which was performed using positive ESI mode. As a consequence of the collision-induced cleavage in the ureido bond of the AMQ adduct of each amino acid or aminothiol, the preferential MRM transition obtained for the different amino acids or aminothiols fraction AMQ (171 +)^52,56^.

#### Sugars

Fructose, glucose, maltose, and sucrose were extracted and analysed with the method by Nadezdha et al.^57^. We finely grounded 2-g samples in a lab homogenizer (Ika, Germany) with 40 mL freeze ethanol (60%, v/v). Samples were weighted, placed and covered with Parafilm on the top of Erlenmeyer flasks in a shaking water bath at 83 °C for 20 min. Samples were then cooled at room temperature and their weigh was brought to the initial by 60% (v/v) ethanol addition and filtered through Whatmann N4 paper. Additional sample clean-up was performed on the filtrate by sequentially filtering through a Sep Pak C18 cartridge (Waters, Milford, MA) and 0.22 µm membrane filter (Sterivex-TM GS, Millipore) before injection in HPLC. Duplicate analysis was carried out on the individual extractions. The HPLC optimisation followed Nadezdha et al. (2013). Chromatographic separation was carried out on HPLC apparatus Hewlet-Packard 1100 series consisted of degasser iso pump, and automatic sample injector. Refractive index detector (RID) Hewlet-Packard 1100 series was used for sugar detection and quantification. The HPLC separation of sugars was performed on Agilent Zorbax Carbohydrate Analysis—aminopropyl column (stationary phase silica spherical particles, 5 µm diameter which is reacted with 3-aminopropyl silane) 4.6 mm × 250 mm. The temperature of the column controlled by a thermostat Agilent 1100 and the RID temperature were both set at 30 °C. We used acetonitrile–water in ratio 80:20 (v/v) at a flow of 2 mL min^−1^ as mobile phase. The injection volume was adjusted to 5 µL. Agilent Chem Station software was used for the control of the system, data acquisition and analysis. Sugar concentration was calculated based on peak area measurements using authentic markers.

### Statistical analyses

*Plant traits* were analysed with a linear mixed model (LMM) where the treatments nitrogen, [CO_2_] and their interaction were fixed effects and replicates of the treatment combinations were incorporated into the LMM as random effects. Wald statistics were used to derive *p*-values of the main and interaction fixed effects. We report *p* as a continuous value, complemented with the Shannon information transformation [s = − log_2_(*p*)] as a measure of information against the tested hypothesis^58^.

*EPG variables* describing aphid feeding were calculated using the EPG-Excel Data Workbook developed by Sarria et al.^59^. EPG variables were calculated as follows: proportion of individuals that produce a specific waveform: the number of insects that produce a specific waveform divided by the total number of insects per treatment; the number of waveform events for each insect; the total waveform duration for each insect calculated as the total duration of a waveform, summed over all occurrences of the waveform for each insect. If no event occurred for a particular insect, then the value considered was 0; and mean duration of waveform events for each insect: the average waveform event duration (total waveform duration divided by the number of waveform events) for each insect. If there was no event for a particular waveform, then the value considered was missing data. The output given by the Sarria et al. (2009) workbook for each replicate was used to calculate the treatment mean for each variable. After extracting EPG variables with this procedure, we analysed data in three ways. First, all EPG raw data were checked for normality and homogeneity of variance using Kolmogorov Smirnoff test; data were log or square root transformed as needed to reduce heteroscedasticity. For each aphid species, we performed pair-wise comparisons of treatments with a Mann–Whitney U-test, for non-parametric variables, or a Student’s t-test for parametric variables (Supplementary Tables 2 and 3). Second, we performed a discriminant analysis to capture the effects of the full combination of experimental sources of variation (nitrogen, [CO_2_], aphid species) on the aphid multivariate EPG waveform data. Initially, a quality assessment of the all EPG variables was undertaken. Due to the small number of non-zero measurements, the G and F waveforms were omitted from this discrimination analysis. For the remaining E and C waveforms, if an EPG variable was a function of other variables it was also omitted to ensure a set of uniquely independent variables was used. The remaining 19 EPG variables were then independently tested for normality using a simple analysis of variance and, where appropriate, a log or square root transformation was performed. With this set of non-redundant variables, a single set of treatments was generated from the factorial combinations of nitrogen, [CO_2_], aphid, and a linear discrimination analysis was conducted^60^ to determine whether EPG variation differed among the levels of the treatment. This method finds an optimal set of linear discriminants (linear combinations of the variables) using a modern Bayes discriminant rule that maximises separation of treatment classes. Similar to principal components analysis, the linear discriminants are ranked by importance. We used the first two linear discriminants to summarise the results. Third, we focused on the biologically significant E2 representing passive phloem feeding (Fig. 6). We plotted the proportion of aphids at E2 as a function of time (30-min intervals during the 8 h assay) as in Garzo et al.^61^. We fitted curves for each combination of nitrogen, [CO_2_] and aphid and used an F-test for comparison of fitted curves^62^.

## Supplementary Information

Supplementary Information 1.
