# Supplementary material for: The role of plant labile carbohydrates and nitrogen on wheat-aphid relations
Source: Sci Rep. 2021 Jun 15;11:12529. doi: 10.1038/s41598-021-91424-8 (PMC8206072; doi:10.1038/s41598-021-91424-8)
Supplement: Supplementary file 1 — Supplementary Information 1. [file 41598_2021_91424_MOESM1_ESM.docx]

Supplementary Table 1. Effect of nitrogen, [CO_2_] and their interaction on the concentration of sugars and amino acids of wheat plants at 28 days after sowing, immediately before the establishment of aphid assays 1 and 2. *p* is from linear mixed model coloured according to the scale below; *s* is the Shannon information transform ^54^ .

| Trait | Source of variation | |  |  |  |  |
| --- | --- | --- | --- | --- | --- | --- |
|  | N |  | C0_2_ |  | interaction | |
|  | *p* | *s* | *p* | *s* | *p* | *s* |
| Total sugars | 0.000 | 50.2 | 0.000 | 21.2 | 0.069 | 3.9 |
| Total amino acids | 0.039 | 4.7 | 0.259 | 2.0 | 0.524 | 0.9 |
| Sugar:amino acid | 0.000 | 27.2 | 0.028 | 5.2 | 0.364 | 1.5 |
|  |  |  |  |  |  |  |
| Fructose | 0.000 | 53.0 | 0.000 | 23.2 | 0.010 | 6.7 |
| Glucose | 0.000 | 21.7 | 0.001 | 10.7 | 0.935 | 0.1 |
| Maltose | 0.000 | 13.7 | 0.082 | 3.6 | 0.421 | 1.2 |
|  |  |  |  |  |  |  |
| Aminobutyric acid | 0.000 | 19.4 | 0.091 | 3.5 | 0.199 | 2.3 |
| Leucine | 0.000 | 13.1 | 0.978 | 0.0 | 0.182 | 2.5 |
| Phenylananine | 0.000 | 11.6 | 0.755 | 0.4 | 0.246 | 2.0 |
| Valine | 0.000 | 11.4 | 0.881 | 0.2 | 0.207 | 2.3 |
| Arginine | 0.001 | 9.8 | 0.370 | 1.4 | 0.999 | 0.0 |
| Serine | 0.001 | 9.5 | 0.500 | 1.0 | 0.004 | 8.1 |
| Threonine | 0.002 | 9.3 | 0.710 | 0.5 | 0.084 | 3.6 |
| Proline | 0.012 | 6.4 | 0.001 | 11.1 | 0.992 | 0.0 |
| Alanine | 0.015 | 6.1 | 0.434 | 1.2 | 0.286 | 1.8 |
| Hydroxyproline | 0.034 | 4.9 | 0.314 | 1.7 | 0.834 | 0.3 |
| Methylhistidine | 0.088 | 3.5 | 0.201 | 2.3 | 0.438 | 1.2 |
| Tryptophane | 0.207 | 2.3 | 0.030 | 5.1 | 0.590 | 0.8 |
| Aspartic acid | 0.219 | 2.2 | 0.023 | 5.4 | 0.654 | 0.6 |
| Tyrosine | 0.389 | 1.4 | 0.767 | 0.4 | 0.151 | 2.7 |
| Glutamic acid | 0.718 | 0.5 | 0.130 | 2.9 | 0.653 | 0.6 |
| Glycine | 0.870 | 0.2 | 0.488 | 1.0 | 0.497 | 1.0 |

Supplementary Table 2. EPG traits for the probing and feeding behaviour of *Rhopalosiphum padi* on wheat plants grown in a factorial with two nitrogen levels and two [CO_2_]. *p*-values are for pair-wise comparisons from t-test for parametric variables and from Mann–Whitney U test for non-Gaussian distribution variables. Shannon information transform is shown between brackets.

| Trait | Nitrogen | | CO_2_ = 700 ppm | | CO_2_ = 400 ppm | | | *p* |
| --- | --- | --- | --- | --- | --- | --- | --- | --- |
|  |  | | Proportion | Mean ± SE | Proportion | | Mean ± SE |  |
| No probe (np) |  | |  |  |  | |  |  |
| Number of np | High | | 17/17 | 11.1 ± 1.74 | 18/18 | | 15.3 ± 1.89 | *0.075 [3.7]* |
|  | Low | | 18/18 | 9.9 ± 1.55 | 16/16 | | 10.9 ± 1.96 | *0.982 [0.0]* |
|  |  | |  | *p=0.909 [0.1]* |  | | *p=0.057 [4.1]* |  |
| Total duration of np (s) | High | | 17/17 | 5668 ± 1213.3 | 18/18 | | 5238 ± 1079.1 | *0.982 [0.0]* |
|  | Low | | 18/18 | 2490 ± 553.3 | 16/16 | | 2729 ± 614.2 | *0.746 [0.4]* |
|  |  | |  | *p= 0.030 [5.1]* |  | | *p=0.055 [4.2]* |  |
| Mean duration of np (s) | High | | 17/17 | 629 ± 129.7 | 18/18 | | 354 ± 58.9 | *0.102 [3.3]* |
|  | Low | | 18/18 | 243 ± 55.7 | 16/16 | | 277 ± 37.9 | *0.206 [2.3]* |
|  |  | |  | *p=0.009 [6.8]* |  | | *p=0.522 [0.9]* |  |
| Probing (tissue penetration) |  | |  |  |  | |  |  |
| Number of probes | High | | 17/17 | 10.5 ± 1.77 | 18/18 | | 15.0 ± 1.84 | *0.051 [4.3]* |
|  | Low | | 18/18 | 9.9 ± 1.55 | 16/16 | | 10.6 ± 1.90 | *0.982 [0.0]* |
|  |  | |  | *p=0.882 [0.2]* |  | | *p=0.056 []* |  |
| Total probing time (s) | High | | 17/17 | 23131 ± 1213.3 | 18/18 | | 23561 ± 1079.1 | *0.909 [0.1]* |
|  | Low | | 18/18 | 23609 ± 553.3 | 16/16 | | 25857 ± 591.6 | *0.746 [0.4]* |
|  |  | |  | *p=0.909 [0.1]* |  | | *p=0.088 [4.2]* |  |
| Time to 1st probe from  start of EPG (s) | High | | 17/17 | 749 ± 317.3 | 18/18 | | 358.4 ± 46.5 | *0.590 [0.8]* |
|  | Low | | 18/18 | 262 ± 35.7 | 16/16 | | 373.1 ± 107.8 | *0.507 [1.0]* |
|  |  | |  | *p=0.590 [0.8]* |  | | *p=0.707 [0.5]* |  |
| Pathway (C) |  | |  |  |  | |  |  |
| Number of C | High | | 17/17 | 17.3 ± 2.46 | 18/18 | | 28.2 ± 2.92 | *0.007 [7.2]* |
|  | Low | | 18/18 | 21.4 ± 2.55 | 16/16 | | 25.4 ± 4.12 | *0.849 [0.2]* |
|  |  | |  | *p=0.066 [3.9]* |  | | *p=0.446 [1.2]* |  |
| Total duration of C (s) | High | | 17/17 | 6675 ± 786.1 | 18/18 | | 12120 ± 1192.2 | *0.001 [10.0]* |
|  | Low | | 18/18 | 9584 ± 995.5 | 16/16 | | 10546 ± 1436.9 | *0.736 [0.4]* |
|  |  | |  | *p=0.029 [5.1]* |  | | *p=0.319 [1.6]* |  |
| Mean duration of C (s) | High | | 17/17 | 450 ± 63.1 | 18/18 | | 469 ± 50.4 | *0.424 [1.2]* |
|  | Low | | 18/18 | 470 ± 29.3 | 16/16 | | 663 ± 221.5 | *0.365 [1.5]* |
|  |  | |  | *p=0.769 [0.4]* |  | | *p=0.528 [0.9]* |  |
| % of probing time in C | High | | 17/17 | 31.7 ± 5.30 | 18/18 | | 52.4 ± 4.7 | *0.006 [7.4]* |
|  | Low | | 18/18 | 37.2 ± 4.06 | 16/16 | | 42.4 ± 6.2 | *0.627 [0.7]* |
|  |  | |  | *p=0.411 [1.3]* |  | | *p=0.161 [2.6]* |  |
| Phloem salivation (E1) |  | |  |  |  | |  |  |
| Number of E1 | High | | 16/17 | 6.4 ± 1.14 | 18/18 | | 12.2 ± 2.1 | *0.043 [1.2]* |
|  | Low | | 18/18 | 10.4 ± 1.73 | 15/16 | | 14.8 ± 2.8 | *0.363 [1.5]* |
|  |  | |  | *p=0.011 [3.2]* |  | | *p=0.534 [0.9]* |  |
| Number of single E1 | High | | 2/17 | 0.29 ± 0.24 | 13/18 | | 2.00 ± 0.52 | *0.001 [10.0]* |
|  | Low | | 9/18 | 1.17 ± 0.40 | 8/16 | | 1.74 ± 0.52 | *0.851 [0.2]* |
|  |  | |  | *p=0.001 [10.0]* |  | | *p=0.297 [1.8]* |  |
| Total duration of E1 (s) | High | | 16/17 | 181 ± 45.8 | 18/18 | | 505 ± 130.66 | *0.025 [5.3]* |
|  | Low | | 18/18 | 538 ± 187.8 | 15/16 | | 715 ± 175.21 | *0.966 [0.0]* |
|  |  | |  | *p=0.007 [7.2]* |  | | *p=0.867 [0.2]* |  |
| Total duration of single E1 (s) | High N | | 2/17 | 7.9 ± 5.6 | 13/18 | | 82.2 ± 21.4 | *0.001 [10.0]* |
|  | Low N | | 9/18 | 46.1 ± 18.4 | 8/16 | | 152.9 ± 86.1 | *0.772 [0.4]* |
|  |  | |  | *p=0.001 [10.0]* |  | | *p=0.281 [1.8]* |  |
| Mean duration of E1 (s) | High | | 16/17 | 32.5 ± 6.03 | 18/18 | | 40.3 ± 8.14 | *0.721 [0.5]* |
|  | Low | | 18/18 | 50.1 ± 10.37 | 15/16 | | 56.7 ± 18.52 | *0.789 [0.3]* |
|  |  | |  | *p=0.151 [2.7]* |  | | *p=0.325 [1.6]* |  |
| Time from 1st probe to 1st E (s) | High | | 17/17 | 3802 ± 1292.28 | 18/18 | 3957 ± 1097.85 | | *0.807 [0.3]* |
|  | Low | | 18/18 | 4249 ± 616.92 | 15/16 | 4534 ± 1569.67 | | *0.507 [1.0]* |
|  |  | |  | *p=0.556 [0.8]* |  | *p=0.767 [0.4]* | |  |
|  |  | |  |  |  |  | |  |
| Probing time spent in E1 (%) | High | | 16/17 | 0.7 ± 0.17 | 18/18 | 2.03 ± 0.50 | | *0.029 [5.1]* |
|  | Low | | 18/18 | 2.0 ± 0.67 | 15/16 | 2.77 ± 0.65 | | *0.446 [1.2]* |
|  |  | |  | *p=0.029 [5.1]* |  | *p=0.596 [0.7]* | |  |
| Contribution of E1 to phloem phase (%) | High | | 16/17 | 1.3 ± 0.31 | 18/18 | 10.8 ± 2.74 | | *0.0001 [ 13.3]* |
|  | Low | | 18/18 | 6.5 ± 1.90 | 15/16 | 10.0 ± 2.93 | | *0.442 [1.2]* |
|  |  | |  | *p=0.0001 [13.3]* |  | *p=0.718 [0.5]* | |  |
| Passive phloem sap ingestion (E2) | |  | |  |  |  | |  |
| Number of E2 | | High | 16/17 | 6.1 ± 1.00 | 18/18 | 10.2 ± 1.78 | | *0.055 [4.2]* |
|  |  | Low | 18/18 | 9.2 ± 1.39 | 15/16 | 13.3 ± 2.65 | | *0.367 [1.4]* |
|  |  |  |  | *p=0.080 [3.6]* |  | *p=0.522 [0.9]* | |  |
| Number of sustained E2 (longer than 10 min) | | High | 16/17 | 1.9 ± 0.23 | 16/18 | 1.4 ± 0.23 | | *0.134 [2.9]* |
|  |  | Low | 16/18 | 1.8 ± 0.27 | 14/16 | 1.9 ± 0.30 | | *0.959 [0.1]* |
|  |  |  |  | *p=0.134 [2.9]* |  | *p=0.330 [1.6]* | |  |
| Total duration of E2 (s) | | High | 16/17 | 14674 ± 1494.7 | 18/18 | 8033 ± 1414.9 | | *0.003 [8.3]* |
|  |  | Low | 18/18 | 11922 ± 1727.6 | 15/16 | 11498 ± 2128.2 | | *0.974 [0.0]* |
|  |  |  |  | *p=0.237 [2.1]* |  | *p=0.140 [2.8]* | |  |
| Mean duration of E2 (s) | | High | 16/17 | 5804 ± 317.3 | 18/18 | 1789 ± 671.5 | | *0.001 [9.7]* |
|  |  | Low | 18/18 | 2909 ± 1305.2 | 15/16 | 2350 ± 915.1 | | *0.700 [0.5]* |
|  |  |  |  | *p=0.003 [8.4]* |  | *p=0.520 [0.9]* | |  |
| Duration of the longest E2 (s) | | High | 16/17 | 12505 ± 1527.9 | 18/18 | 6139 ± 1382.4 | | *0.004 [7.9]* |
|  |  | Low | 18/18 | 9445 ± 1737.7 | 15/16 | 8481 ± 915.1 | | *0.876 [0.2]* |
|  |  |  |  | *p=0.031 [5.0]* |  | *p=0.322 [1.6]* | |  |
| % of probing time spent in E2 | | High | 16/17 | 60.7 ± 5.36 | 18/18 | 32.6 ± 5.2 | | *0.001 [9.8]* |
|  |  | Low | 18/18 | 44.6 ± 5.95 | 15/16 | 42.9 ± 7.5 | | *0.984 [0.0]* |
|  |  |  |  | *p=0.053 [4.2]* |  | *p=0.198 [2.3]* | |  |
| % of sustained E2 | | High | 16/17 | 47.7 ± 8.39 | 16/18 | 24.7 ± 6.92 | | *0.015 [6.0]* |
|  |  | Low | 16/18 | 28.7 ± 6.24 | 14/16 | 21.2 ± 4.34 | | *0.508 [1.0]* |
|  |  |  |  | *p=0.003 [8.4]* |  | *p=0.999 [0.0]* | |  |
| Potential E2 index | | High | 16/17 | 60.6 ± 4.85 | 18/18 | 32.2 ± 5.50 | | *0.000 [13.3]* |
|  |  | Low | 18/18 | 48.9 ± 6.51 | 15/16 | 47.3 ± 8.03 | | *0.958 [0.1]* |
|  |  |  |  | *p=0.161 [2.6]* |  | *p=0.093 [3.4]* | |  |
| Time from 1st probe to 1st sustained E2 (s) | | High | 16/17 | 4795 ± 1349.0 | 16/18 | 8147 ± 2169.5 | | *0.275 [1.9]* |
|  |  | Low | 16/18 | 9189 ± 1888.1 | 14/16 | 9070 ± 2191.3 | | *0.707 [0.5]* |
|  |  |  |  | *p=0.032 [5.0]* |  | *p=0.596 [0.7]* | |  |
| Time from the beginning of that probe to 1st sustained E2 (s) | | High | 16/17 | 1726 ± 440.1 | 16/18 | 1504 ± 20.5 | | *0.938 [0.1]* |
|  |  | Low | 16/18 | 2431 ± 628.5 | 14/16 | 1749 ± 314.2 | | *0.638 [0.6]* |
|  |  |  |  | *p=0.606 [0.7]* |  | *p=0.481 [1.1]* | |  |

Supplementary Table 3. EPG traits for the probing and feeding behaviour of *Sitobion avenae* on wheat plants grown in a factorial with two nitrogen levels and two [CO_2_]. *p*-values are for pair-wise comparisons from t-test for parametric variables and from Mann–Whitney U test for non-Gaussian distribution variables. Shannon *s*-value is shown between brackets.

| Trait | Nitrogen | | CO_2_ = 700 ppm | | CO_2_ = 400 ppm | | | *p* |
| --- | --- | --- | --- | --- | --- | --- | --- | --- |
|  |  | | Proportion | Mean ± SE | Proportion | | Mean ± SE |  |
| No probe (np) |  | |  |  |  | |  |  |
| Number of np | High | | 19/19 | 7.9 ± 1.05 | 17/17 | | 7.7 ± 0.62 | *0.882 [0.2]* |
|  | Low | | 17/17 | 5.9 ± 0.92 | 19/19 | | 12.7 ± 1.39 | *0.0001 [13.3]* |
|  |  | |  | *p=0.171 [2.5]* |  | | *p=0.009 [6.8]* |  |
| Total duration of np (s) | High | | 19/19 | 2742 ± 534.7 | 17/17 | | 5954 ± 1144.5 | *0.024 [5.4]* |
|  | Low | | 17/17 | 3561 ± 1127.8 | 19/19 | | 7858 ± 1312.2 | *0.021 [5.6]* |
|  |  | |  | *p=0.866 [0.2]* |  | | *p=0.258 [2.0]* |  |
| Mean duration of np (s) | High | | 19/19 | 407 ± 125.6 | 17/17 | | 764 ± 150.5 | *0.030 [5.1]* |
|  | Low | | 17/17 | 630 ± 247.8 | 19/19 | | 661 ± 109.2 | *0.147 [2.8]* |
|  |  | |  | *p=0.713 [0.5]* |  | | *p=0.882 [0.2]* |  |
| Probing (tissue penetration) |  | |  |  |  | |  |  |
| Number of probes | High | | 19/19 | 7.7 ± 1.06 | 17/17 | | 7.2 ± 0.59 | *0.691 [0.5]* |
|  | Low | | 17/17 | 5.7 ± 0.88 | 19/19 | | 12.5 ± 1.38 | *0.0001 [13.3]* |
|  |  | |  | *p=0.149 [2.7]* |  | | *p=0.009 [6.8]* |  |
| Total probing time (s) | High | | 19/19 | 26058 ± 534.7 | 17/17 | | 22835 ± 1148.5 | *0.049 [4.4]* |
|  | Low | | 17/17 | 25239 ± 1127.8 | 19/19 | | 20942 ± 1312.2 | *0.003 [8.4]* |
|  |  | |  | *p=0.827 [0.3]* |  | | *p=0.261 [1.9]* |  |
| Time to 1st probe from  start of EPG (s) | High | | 19/19 | 179 ± 43.4 | 16/17 | | 710 ± 321.8 | *0.208 [2.3]* |
|  | Low | | 17/17 | 383 ± 167.4 | 19/19 | | 389 ± 107.0 | *0.298 [1.7]* |
|  |  | |  | *p=0.942 [0.1]* |  | | *p=0.829 [0.3]* |  |
| Pathway (C) |  | | 19/19 | 7.74 ± 1.06 | 17/17 | | 7.24 ± 0.59 | *0.691* |
| Number of C | High | | 19/19 | 14.0 ± 1.9 | 17/17 | | 12.4 ± 1.2 | *0.501 [1.0]* |
|  | Low | | 17/17 | 10.3 ± 1.1 | 19/19 | | 18.1 ± 1.9 | *0.001 [10.0]* |
|  |  | |  | *p=0.111 [3.2]* |  | | *p=0.045 [4.5]* |  |
| Total duration of C (s) | High | | 19/19 | 10195 ± 1183.5 | 17/17 | | 11621 ± 1041.1 | *0.372 [1.4]* |
|  | Low | | 17/17 | 10412 ± 1720.5 | 19/19 | | 11733 ± 1293.6 | *0.544 [0.9]* |
|  |  | |  | *p=0.918 [0.1]* |  | | *p=0.774 [0.4]* |  |
| Mean duration of C (s) | High | | 19/19 | 884 ± 130.9 | 17/17 | | 987 ± 88.8 | *0.129 [3.0]* |
|  | Low | | 17/17 | 1188 ± 297.4 | 19/19 | | 680 ± 67.0 | *0.175 [2.5]* |
|  |  | |  | *p=0.427 [1.2]* |  | | *p=0.005 [7.6]* |  |
| % of probing time in C | High | | 19/19 | 39.3 ± 4.58 | 17/17 | | 52.5 ± 4.48 | *0.048 [4.4]* |
|  | Low | | 17/17 | 41.3 ± 6.24 | 19/19 | | 59.7 ± 6.08 | *0.049 [4.4]* |
|  |  | |  | *p=0.796 [0.3]* |  | | *p=0.160 [2.6]* |  |
| Phloem salivation (E1) |  | |  |  |  | |  |  |
| Number of E1 | High | | 19/19 | 6.0 ± 1.19 | 17/17 | | 4.7 ± 0.87 | *0.452 [1.1]* |
|  | Low | | 15/17 | 3.8 ± 0.75 | 17/19 | | 4.8 ± 0.96 | *0.918 [0.1]* |
|  |  | |  | *p=0.130 [2.9]* |  | | *p=0.567 [0.8]* |  |
| Number of single E1 | High | | 10/19 | 1.3 ± 0.33 | 10/17 | | 0.9 ± 0.25 | *0.707 [0.5]* |
|  | Low | | 9/17 | 1.1 ± 0.30 | 13/19 | | 1.7 ± 0.46 | *0.346 [1.5]* |
|  |  | |  | *p=0.778 [0.4]* |  | | *p=0.518 [0.9]* |  |
| Total duration of E1 (s) | High | | 19/19 | 1017 ± 186.3 | 17/17 | | 897 ± 148.5 | *0.617 [0.7]* |
|  | Low | | 15/17 | 632 ± 146.7 | 17/19 | | 897 ± 193.9 | *0.648 [0.6]* |
|  |  | |  | *p=0.088 [3.5]* |  | | *p=0.705 [0.5]* |  |
| Total duration of single E1 (s) | High N | | 10/19 | 167 ± 53.5 | 10/17 | | 118 ± 51.6 | *0.827 [0.3]* |
|  | Low N | | 9/17 | 175 ± 54.7 | 13/19 | | 237 ± 65.2 | *0.510 [1.0]* |
|  |  | |  | *p=0.876 [0.2]* |  | | *p=0.433 [1.2]* |  |
| Mean duration of E1 (s) | High | | 19/19 | 289 ± 75.0 | 17/17 | | 211 ± 33.8 | *0.968 [0.0]* |
|  | Low | | 15/17 | 183 ± 37.7 | 17/19 | | 171 ± 25.5 | *0.858 [0.2]* |
|  |  | |  | *p=0.594 [0.8]* |  | | *p=0.386 [1.4]* |  |
| Time from 1st probe to 1st E (s) | High | | 19/19 | 6439 ± 1014.99 | 17/17 | 6538 ± 1274.5 | | *0.960 [0.1]* |
|  | Low | | 15/17 | 10270 ± 2333.5 | 16/19 | 11702 ± 2208.0 | | *0.619 [0.7]* |
|  |  | |  | *p=0.261 [1.9]* |  | *p=0.093 [3.4]* | |  |
|  |  | |  |  |  |  | |  |
| Probing time spent in E1 (%) | High | | 19/19 | 3.86 ± 0.69 | 17/17 | 3.98 ± 0.66 | | *0.901 [0.2]* |
|  | Low | | 15/17 | 2.34 ± 0.66 | 17/19 | 4.13 ± 0.85 | | *0.426 [1.2]* |
|  |  | |  | *p=0.167 [2.6]* |  | *p=0.828 []* | |  |
| Contribution of E1 to phloem phase (%) | High | | 19/19 | 9.93 ± 2.43 | 17/17 | 15.63 ± 3.89 | | *0.219 [2.2]* |
|  | Low | | 15/17 | 18.03 ± 7.06 | 17/19 | 36.56 ± 7.29 | | *0.064 [4.0]* |
|  |  | |  | *p=0.812 [0.3]* |  | *p=0.085 [0.3]* | |  |
| Passive phloem sap ingestion (E2) | |  | |  |  |  | |  |
| Number of E2 | | High | 19/19 | 4.7 ± 1.02 | 17/17 | 3.7 ± 0.77 | | *0.588 [0.8]* |
|  |  | Low | 14/17 | 2.7 ± 0.58 | 16/19 | 3.0 ± 0.64 | | *0.917 [0.1]* |
|  |  |  |  | *p=0.084 [3.6]* |  | *p=0.198 [3.6]* | |  |
| Number of sustained E2 (longer than 10 min) | | High | 18/19 | 1.5 ± 0.21 | 15/17 | 1.4 ± 0.19 | | *0.925 [0.5]* |
|  |  | Low | 14/17 | 1.5 ± 0.31 | 10/19 | 0.6 ± 0.14 | | *0.021 [5.6]* |
|  |  |  |  | *p=0.778 [0.4]* |  | *p=0.009 [6.8]* | |  |
| Total duration of E2 (s) | | High | 19/19 | 13116 ± 1436.5 | 17/17 | 8662 ± 1477.7 | | *0.038 [4.7]* |
|  |  | Low | 14/17 | 10833 ± 2406.9 | 16/19 | 6018 ± 1911.4 | | *0.163 [2.6]* |
|  |  |  |  | *p=0.410 [1.3]* |  | *p=0.125 [3.0]* | |  |
| Mean duration of E2 (s) | | High | 19/19 | 7040 ± 1865.1 | 17/17 | 3552 ± 799.2 | | *0.134 [2.9]* |
|  |  | Low | 14/17 | 6964 ± 2316.6 | 16/19 | 4482 ± 1894.6 | | *0.136 [2.9]* |
|  |  |  |  | *p=0.990 [0.0]* |  | *p=0.981 [0.0]* | |  |
| Duration of the longest E2 (s) | | High | 19/19 | 11219 ± 1710.5 | 17/17 | 6757 ± 1269.9 | | *0.058 [4.1]* |
|  |  | Low | 14/17 | 11050 ± 2469.4 | 16/19 | 6593 ± 2197.5 | | *0.192 [2.4]* |
|  |  |  |  | *p=0.795 [0.3]* |  | *p=0.624 [0.7]* | |  |
| % of probing time spent in E2 | | High | 19/19 | 50.3 ± 5.41 | 17/17 | 36.2 ± 5.30 | | *0.072 [3.8]* |
|  |  | Low | 14/17 | 41.3 ± 8.33 | 16/19 | 24.3 ± 7.29 | | *0.163 [2.6]* |
|  |  |  |  | *p=0.362 [1.5]* |  | *p=0.116 [3.1]* | |  |
| % of sustained E2 | | High | 18/19 | 54.6 ± 8.66 | 15/17 | 50.2 ± 8.35 | | *0.950 [0.1]* |
|  |  | Low | 14/17 | 67.7 ± 9.11 | 10/19 | 30.7 ± 9.33 | | *0.006 [7.4]* |
|  |  |  |  | *p=0.397 [1.3]* |  | *p=0.143 [2.8]* | |  |
| Potential E2 index | | High | 19/19 | 64.7 ± 6.55 | 17/17 | 43.6 ± 6.71 | | *0.031 [5.0]* |
|  |  | Low | 14/17 | 62.3 ± 8.45 | 16/19 | 38.0 ± 9.90 | | *0.093 [3.4]* |
|  |  |  |  | *p=0.928 [0.1]* |  | *p=0.934 [0.1]* | |  |
| Time from 1st probe to 1st sustained E2 (s) | | High | 19/19 | 11338 ± 1574.2 | 15/17 | 12249 ± 1985.2 | | *0.722 [0.5]* |
|  |  | Low | 14/17 | 12718 ± 2386.8 | 10/19 | 19959 ± 2398.3 | | *0.076 [3.7]* |
|  |  |  |  | *p=0.900 [0.2]* |  | *p=0.120 [3.1]* | |  |
| Time from the beginning of that probe to 1st sustained E2 (s) | | High | 18/19 | 2651 ± 454.5 | 15/17 | 3457 ± 561.8 | | *0.274 [1.9]* |
|  |  | Low | 14/17 | 3202 ± 767.4 | 10/19 | 2272 ± 381.5 | | *0.347 [1.5]* |
|  |  |  |  | *p=0.543 [0.9]* |  | *p=0.087 [3.5]* | |  |
